# Dystrophin Dp71ab is monoclonally expressed in human satellite cells and enhances proliferation of myoblast cells

**DOI:** 10.1038/s41598-020-74157-y

**Published:** 2020-10-13

**Authors:** Manal Farea, Abdul Qawee Mahyoob Rani, Kazuhiro Maeta, Hisahide Nishio, Masafumi Matsuo

**Affiliations:** 1 Research Center for Locomotion Biology, Kobe Gakuin University, 518 Arise, Ikawadani, Nishi, Kobe, 651-2180 Japan; 2 KNC Department of Nucleic Acid Drug Discovery, Faculty of Rehabilitation, Kobe Gakuin University, 518 Arise, Ikawadani, Nishi, Kobe, 651-2180 Japan; 3 Faculty of Rehabilitation, Kobe Gakuin University, 518 Arise, Ikawadani, Nishi, Kobe, 651-2180 Japan

**Keywords:** Medical research, Molecular medicine, Neurology

## Abstract

Dystrophin Dp71 is the smallest isoform of the *DMD* gene, mutations in which cause Duchenne muscular dystrophy (DMD). Dp71 has also been shown to have roles in various cellular processes. Stem cell-based therapy may be effective in treating DMD, but the inability to generate a sufficient number of stem cells remains a significant obstacle. Although Dp71 is comprised of many variants, Dp71 in satellite cells has not yet been studied. Here, the full-length Dp71 consisting of 18 exons from exons G1 to 79 was amplified by reverse transcription-PCR from total RNA of human satellite cells. The amplified product showed deletion of both exons 71 and 78 in all sequenced clones, indicating monoclonal expression of Dp71ab. Western blotting of the satellite cell lysate showed a band corresponding to over-expressed Dp71ab. Transfection of a plasmid expressing Dp71ab into human myoblasts significantly enhanced cell proliferation when compared to the cells transfected with the mock plasmid. However, transfection of the Dp71 expression plasmid encoding all 18 exons did not enhance myoblast proliferation. These findings indicated that Dp71ab, but not Dp71, is a molecular enhancer of myoblast proliferation and that transfection with Dp71ab may generate a high yield of stem cells for DMD treatment.

## Introduction

The *DMD* gene is one of the largest genes in the human genome, consisting of 79 exons spanning a 14-kb transcript, which encodes the protein dystrophin Dp427. Alternative promoters scattered throughout the *DMD* gene produce at least eight tissue- or development-specific isoforms^1–3^. One alternative downstream promoter located in intron 62 produces a transcript consisting of a unique exon, G1 and *DMD* exons 63–79 shared with other dystrophin isoforms^4,5^. This transcript encodes a nearly 71-kDa protein, dystrophin Dp71, an isoform that is expressed ubiquitously^6^.

Dp71 mRNA is characterized by multiple alternative splicings of exons 71, 71–74, and 78 and intron 77, resulting in the expression of more than10 types of Dp71 splice variants^6–8^. Independent skipping of exons 71 and 78 yields Dp71a and Dp71b, respectively, whereas simultaneous skipping produces Dp71ab^6^. Dp71 plays important roles in various cellular processes, including water homeostasis, nuclear architecture and cell adhesion, as well as in cell division and survival^6,9–11^. Recently, Dp71 has been studied in several types of cancer^12–16^ but the roles of Dp71 in cancer development are still controversial. Although the splice variants of Dp71 have been implicated in specific functions^9,11,17–19^, the functional characterization of each splice variant has been hampered by the co-expression of isoforms^20,21^.

Duchenne muscular dystrophy (DMD) is a fatal progressive muscle wasting disease, caused by mutations in the *DMD* gene^1,22^. Many strategies, such as stem cell-based therapy have been proposed to treat DMD^23^. Satellite cells are muscle stem cells that generate myoblasts by cell division^24^. Myoblasts, in turn, proliferate, differentiate, and fuse to form new myofibers and restore tissue functionality. The regenerative capacity of muscle is diminished in patients with DMD, because errors in cell division exhaust the supply of satellite cells^25–27^. Therefore, therapeutic targets for DMD treatment include satellite cell transplantation and modulation of satellite cell division^24,28^. Although Dp71 has been implicated in cell division^11^, Dp71 has not yet been examined in satellite cells.

The present study therefore assessed the expression of Dp71 in human satellite cells by reverse transcription (RT)-PCR amplification and Western blotting. Dp71ab was shown to be monoclonally expressed in the satellite cells. In addition, over-expression of Dp71ab enhanced myoblast proliferation, suggesting that expression of Dp71ab may generate a high yield of stem cells for DMD treatment.

## Results

### Characterization of gene expression in satellite cells

**Figure 1:**
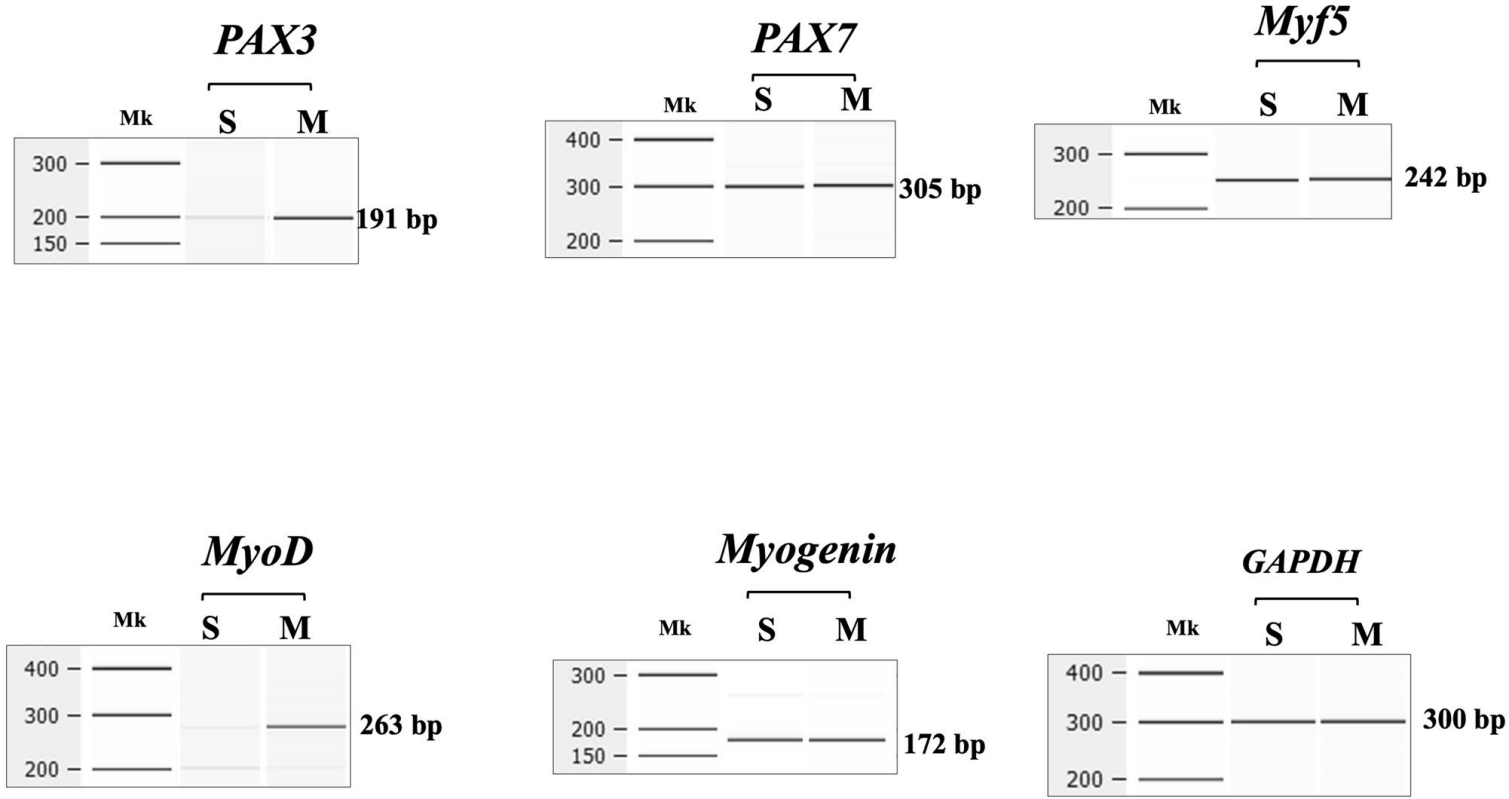
RT-PCR amplification of marker gene mRNAs in human satellite cells. Electropherograms of amplified products are shown. Fragments of the *PAX3*, *PAX7*, *Myf5*, *MyoD*, and *myogenin* genes were amplified from total RNA of satellite cells (S) and skeletal muscle (M). In the satellite cells, expected size product was obtained at different density from *PAX3*, *PAX7*, *Myf5*, and *myogenin*, but not from *MyoD*. In contrast, all 5 fragments were amplified in skeletal muscle (M). The expected size of each amplified product is shown on the right margin of each electropherogram. Mk refers to the 100 bp ladder size marker. Full gel-images are available in Supplementary Figs. 1, 2 and 3.

The expression pattern of some genes in total RNA was first analyzed to characterize the human satellite cells. The expression of mRNAs encoding the *PAX3*, *PAX7*, *Myf5*, *MyoD*, and *myogenin* genes in these cells, as well as in adult skeletal muscle control sample, was analyzed by RT-PCR. In the satellite cells, the amplified products of *PAX7*, *Myf5* and *myogenin* transcripts showed the expected size and the same density as the products from the adult skeletal muscles (Fig. 1). However, the product of *PAX3* was revealed as a weak band compared to adult skeletal muscles. Notably, the product of *MyoD* was revealed as a barely visible band in the satellite cells, while it was clear in skeletal muscle (Fig. 1). It was concluded that the satellite cells used in this study are *PAX7* (+) and *MyoD* (−)^29^.

### Monoclonal expression of Dp71ab in satellite cells

**Figure 2:**
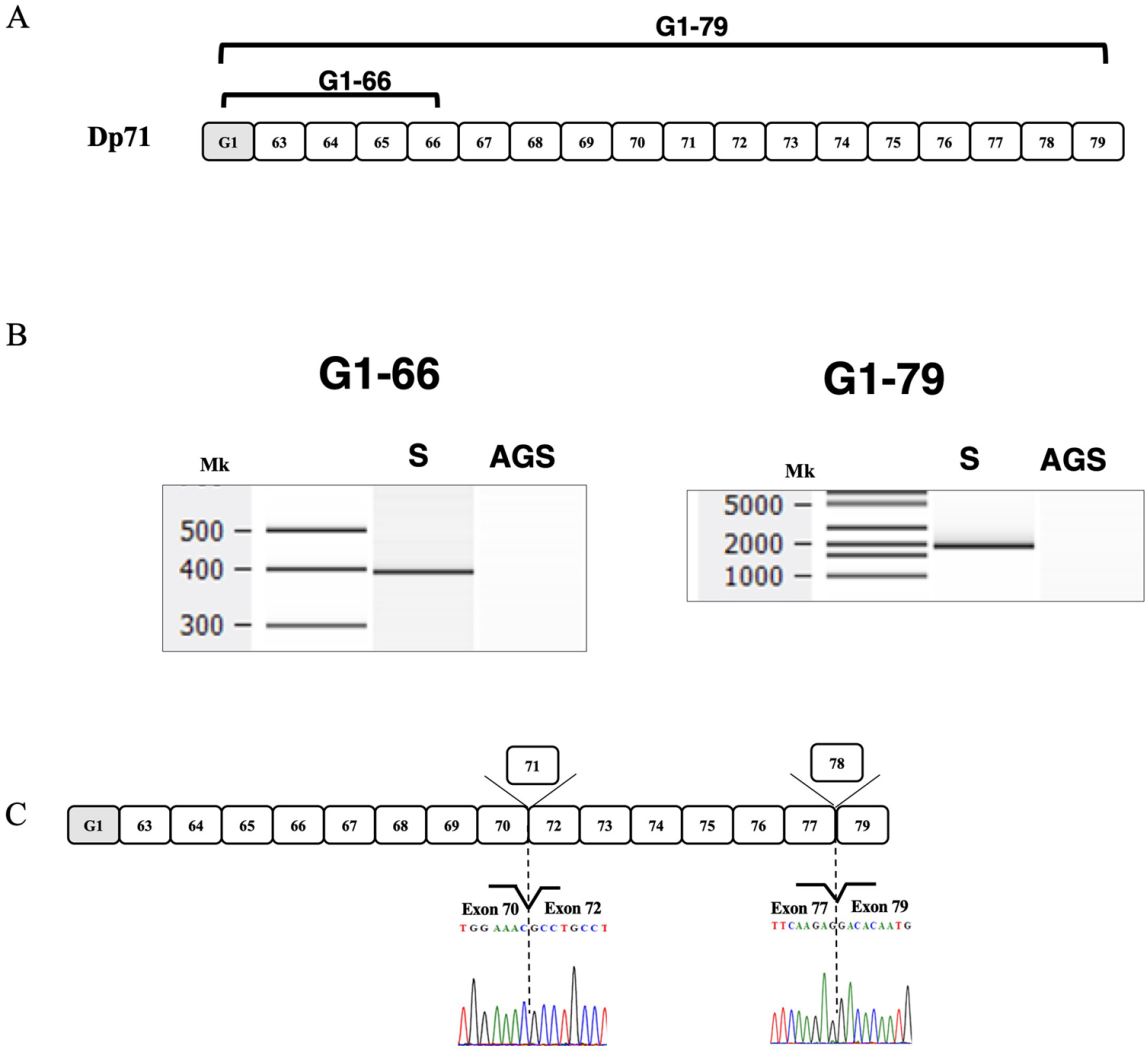
Dp71ab transcript in human satellite cells. (**A**) Schematic description of Dp71 mRNA. The exon structure of Dp71 is shown. Boxes indicate exons. The shaded exon indicates the Dp71-specific exon1 (G1). Numbers in boxes indicate *DMD* exon number. Brackets indicate the amplified fragment. Numbers over the bracket indicate exon number of amplified fragments. (**B**) Amplified product of Dp71 transcript: the 5′ end fragment and full-length Dp71 transcript were RT-PCR amplified from total RNA of human satellite cells. Electropherograms of amplified products are shown. The amplified fragment from exon G1 to exon 66 was of the expected size (lane S in the left panel). Amplification of full-length Dp71, from exon G1 to exon 79, yielded a single product of expected size (lane S in the right panel). In AGS cells, in contrast, both fragments were not obtained (AGS lane in left and right panels). Mk refers to size markers. (**C**) Schematic description of Dp71ab. The exon structure of Dp71ab is shown as in panel (**A**). Boxes and boxes over the bar indicate exons and skipped exons, respectively. Partial sequences of the junctions between exons 70 and 72, and between exons 77 and 79, are shown below these junctions. Full gel-images are available in Supplementary Figs. 4 and 5.

To determine whether the Dp71 promoter was activated in these human satellite cells, an exon G1 covering fragment was PCR amplified using a forward primer complementary to a sequence in exon G1 and a reverse primer complementary to a sequence in exon 66 (Fig. 2A). This amplification yielded a product of expected size (Fig. 2B), indicating Dp71 promoter activation. Because the Dp71 transcript has been reported to consist of more than ten alternative splicing products^7,8^, the full-length Dp71 transcript was amplified using a set of primers on exon G1 and exon 79. This amplification yielded a single band of molecular weight around 2000 bp (Fig. 2B), confirming the expression of Dp71. To determine the exon structure of the product, 30 clones of the full-length Dp71 products were sequenced. Remarkably, all 30 sequenced clones showed deletions of both exons 71 and 78 (Fig. 2C), indicating that a single splicing pattern that removes both of these exons occurred in satellite cells. These findings indicate that Dp71ab is monoclonally expressed in satellite cells.

### Dp71ab protein expression in satellite cells

**Figure 3:**
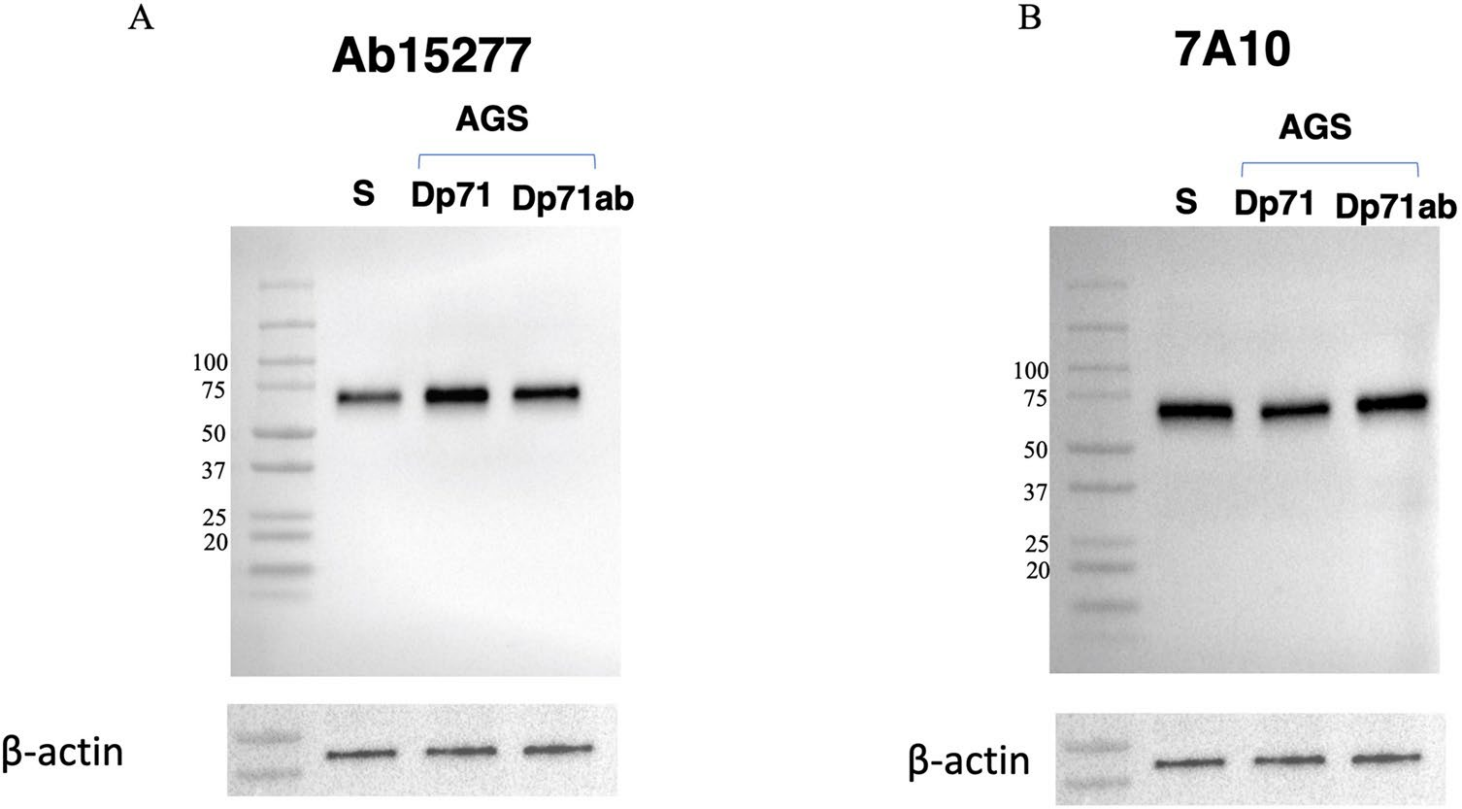
Western blot assay of Dp71ab in human satellite cells. Lysates of human satellite cells and AGS cells transfected with Dp71 and Dp71ab-expression plasmids were analyzed by Western blotting using antibodies ab12577 and 7A10, directed against different domains of the C-terminal region of dystrophin. The immunoblotting results are shown. Binding of ab12577 (**A**) and 7A10 (**B**) antibodies to proteins in AGS cells expressing Dp71 (Dp71) and Dp71ab (Dp71ab) was disclosed. These antibodies also bound to a protein in human satellite cells (S) located at the same position as artificially expressed Dp71ab. The blots were reacted with antibody to β-actin as a loading control (bottom). The left margins show molecular size markers. Full gel-images are available in Supplementary Figs. 6, 7, and 8.

To assess the expression of Dp71ab protein, satellite cell lysates were analyzed by western blotting. As controls, AGS cells, a gastric cancer cell line that does not express endogenous Dp71 (Fig. 2B), were transfected with plasmids expressing either Dp71 or Dp71ab, resulting in the over-expression of each plasmid. Western blotting of lysates of these transfected cells with the anti-Dp71 antibodies ab15277 (Fig. 3A) and 7A10 (Fig. 3B) yielded bands of expected sizes, around 75 kDa, indicating that the proteins encoded by Dp71ab and Dp71 were of similar sizes. Since the satellite cell lysates yielded a protein band of the same size as Dp71ab using both antibodies (Fig. 3A,B), it implied that Dp71ab protein is expressed in human satellite cells.

### Enhancement of myoblast proliferation with Dp71ab

**Figure 4:**
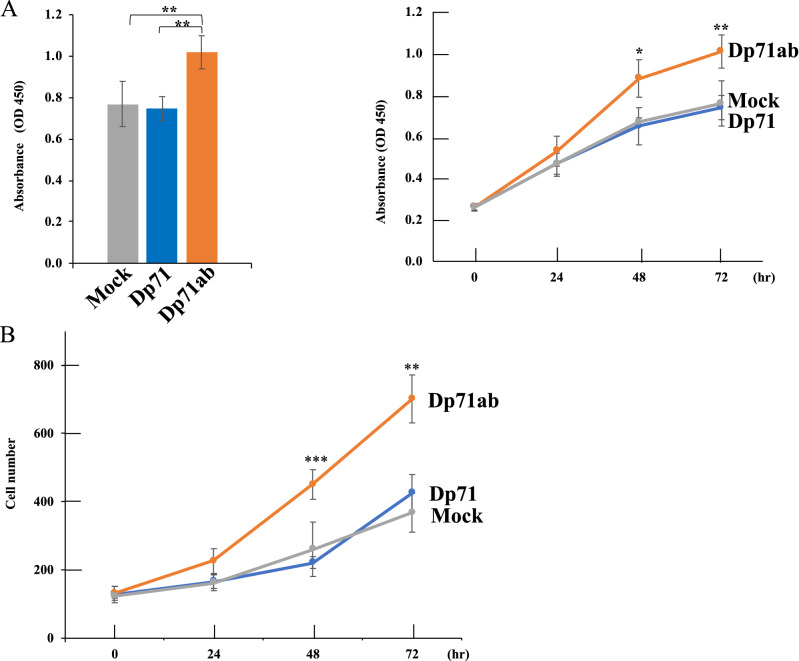
Myoblast cell proliferation. (**A**) CCK-8 assay of the proliferation of myoblasts transfected with the mock-, Dp71- and Dp71ab- plasmids at 72 h (left). The absorbance of cells expressing Dp71ab was significantly higher than the absorbance of the Dp71- or mock-transfected cells (left). Absorbance is expressed as the mean ± standard error of the mean (SEM) of three independent experiments. Time course showing that cell absorbance began to increase significantly from baseline at 48 h (right). *p < 0.05, **p < 0.01. (**B**) Time course of numbers of myoblasts transfected with the mock-, Dp71- and Dp71ab-plasmids from 0 to 72 h. Cells were counted in a microscopic field of each well at 0, 24, 48 and 72 h. Data are expressed as mean ± SEM of three independent experiments. **p < 0.01, ***p < 0.001.

The effect of Dp71ab on the proliferation of human myoblasts was assessed by transfecting these cells with plasmids expressing Dp71ab or Dp71 and analyzing the cell proliferation at 72 h using the CCK-8 assay. The absorbance of the cells transfected with the plasmid expressing Dp71ab (1.02) was significantly higher (p < 0.01) than that of cells transfected with the mock plasmid (0.77) and the plasmid expressing Dp71 (0.75), suggesting that Dp71ab enhanced cell proliferation (Fig. 4A). Analysis of absorbance over time showed that absorbance increased linearly in mock- and Dp71-transfected cells, with no significant difference between them at any time point. The increase in absorbance of Dp71ab-transfected cells was significantly higher, beginning on day 2, indicating that Dp71ab enhanced myoblast proliferation. Direct cell counting using a fluorescence microscope showed that, despite the seeding of the same number of cells in each well, the density of cells transfected with the plasmid expressing Dp71ab was higher than that of the mock- and Dp71-transfected cells (Fig. 4B). Counting of cell numbers over time showed that the numbers of cells on days 2 and 3 were significantly higher following transfection of the plasmid expressing Dp71ab than that of the control plasmid, whereas the number of the Dp71 expressing cells did not differ from the number of mock transfected cells (p < 0.77) (Fig. 4B). These findings indicate that Dp71ab enhances myoblast proliferation.

## Discussion

The present study showed that dystrophin Dp71ab, consisting of Dp71 without exons 71 and 78, was monoclonally expressed in human satellite cells found to be *PAX7* (+) and *MyoD* (−). Monoclonal expression of Dp71ab was unexpected, as Dp71ab transcripts constitute a small fraction of Dp71 transcripts in amniocytes and embryonic kidney cells^21,30^, as well as in rat pheochromocytoma cells^9,31^. Monoclonal expression of Dp71ab was confirmed by nucleotide sequencing of all 30 clones, which showed that all of the amplified products were identical. The alternative splicing demonstrating deletion of both exons 71 and 78 may constitute a default splicing pathway in satellite cells.

Splicing out of *DMD* exon 78 of the Dp427 transcript has been reported to occur between 11 and 18 weeks of gestation, during the development of human skeletal muscle, being almost complete after 20 weeks^32^. Thus, omission of exon 78 may be a default pathway at this embryological stage. Dp71ab has been detected in embryonic cells, including cultured amniocytes^30^ and embryonal kidney cells^21^. Because satellite cells are muscle stem cells, complete omission of exon 78 in satellite cells was not unexpected. In contrast, it is not clear whether skipping of exon 71 is an embryological event. Splicing of exon 78 in the Dp427 transcript is regulated by MBNL1 RNA-binding protein^32^. If splicing of exon 71 is also regulated by MBNL1, then a single mechanism involving the absence of MBNL1 would be responsible for the omission of both exons 71 and 78 from these transcripts.

The deletion of exon 71, which consists of 39 nucleotides, did not alter the reading frame, resulting in the removal of 13 amino acids from the Dp71 protein. In contrast, the deletion of exon 78, which consists of 32 nucleotides, does change the reading frame, replacing the last 13 amino acids with a hydrophobic C-terminus consisting of 31 unique amino acids^30^. Dp71ab consists of 622 amino acids with a molecular weight 70.8 kDa, whereas Dp71 consists of 617 amino acids with a molecular weight of 70.4 kDa. Structurally, the C-terminus of Dp71 is thought to consist of a β-sheet, whereas the C-terminus of Dp71ab is thought to consist of an amphipathic α-helix^32^. Western blotting could not distinguish between Dp71 and Dp71ab. The finding of a single Dp71ab mRNA in satellite cells suggests that this Dp71ab is the only Dp71 protein in satellite cells.

Dp71 downregulation by specific shRNA has been reported to dramatically reduce the proliferation of sarcoma cell lines^15^, strongly suggesting that Dp71 is a driver of cell proliferation. The present study showed that Dp71ab markedly enhanced myoblast proliferation, whereas Dp71 did not. The longer C-terminal sequence of Dp71ab may be responsible in part for enhancing myoblast proliferation. We previously showed that Dp71ab was localized to the nuclei of HEK293 cells^21^. Dp71ab may promote cell division by interacting with β-dystroglycan at the mitotic spindle poles^6^.

Exon skipping therapy using antisense oligonucleotides (ASO) to change the reading frame^33^ may be effective in the treatment of DMD^34^. Although one ASO, eteplirsen, which induces skipping of *DMD* exon 51, was approved for treatment of DMD in 2016^35^, this agent had low clinical efficacy. The efficacy of exon skipping therapy may be improved by enhancing ASO delivery or increasing the number of muscle cells. Stem cell therapy has been tested for the treatment of DMD^23^. For example, an ongoing clinical trial is testing the effects of myoblast transplantation in DMD patients (NCT02196467). In that trial, a sufficient number of myoblasts will be transplanted to sustain the lifetime of transferred cells within the host. The generation of sufficient numbers of transplantable cells remains a significant obstacle to stem cell therapy. To generate a high yield of stem cells with myogenic potential, isolated cells are expanded by culturing with biomaterials^36^. The ability of Dp71ab to enhance myoblast cell proliferation indicates that increased expression of Dp71ab may enhance stem cell proliferation.

## Methods

### Cells

The AGS gastric adenocarcinoma cell line was obtained from the American Type Culture Collection (ATCC; Manassas, VA, USA) and cultured at 37 °C in a 5% CO_2_ humidified incubator in Dulbecco’s modified Eagle Medium (DMEM; Gibco Life Technologies, Waltham, MA, USA), supplemented with 10% fetal bovine serum (FBS; Gibco Life Technologies) and 1% Antibiotic–Antimycotic solution (Gibco Life Technologies). Human myoblasts, generated by the immortalization of primary cultured human myogenic cells, were the kind gift of Dr. Hashimoto^37^. Myoblast cells were grown in DMEM supplemented with 10% FBS and 1% Antibiotic–Antimycotic solution at 37 °C in a humidified 5% CO_2_ incubator.

### mRNA analysis

Cultured cells were rinsed twice with phosphate buffered saline (PBS; FUJIFILM Wako Pure Chemical Corporation, Osaka, Japan) and collected using the lysis/binding buffer in High Pure RNA isolation kits (Roche Diagnostics, Basel, Switzerland). Total RNA was extracted from these cells using High Pure RNA isolation kits (Roche Diagnostics)*.* Total RNAs of human satellite cells and skeletal muscle were obtained from ScienCell Research Laboratories, Inc. (Carlsbad, CA, USA; catalogue no. 3515) and Clontech Laboratories, Inc. (Mountain View, CA, USA; catalogue no. 636534), respectively. cDNA was synthesized from 0.5 µg of total RNA using random primers as described^38^. Human satellite cells were characterized by assessing the expression of paired box transcription factor 3 (*PAX3*), *PAX7*, *Myf5*, *MyoD*, and *myogenin* mRNAs by RT-PCR, using primers that amplified fragments of *PAX3* (forward,: 5′-GTCAACCAGCTCGGCGGTGTTT-3′; reverse, 5′-ATGGCACCAGGACGTATGGGT-3′); *PAX7* (forward, 5′-GGGTCTTCATCAATGGGCGA-3′; reverse, 5′-GTCACAGTGCCCATCCTTCA-3′); *Myf* (forward, 5′-TGGATGGCTGCCAGTTCTCA-3′; reverse, 5′-CCGATCCATGGTGGTGGACT-3′); *MyoD* (forward, 5′-AGCACTACAGCGGCGACT-3′; reverse, 5′-CGACTCAGAAGGCACGTC-3′); and *myogenin* (forward, 5′-CTGCTCAGCTCCCTCAACCA-3′; reverse, 5′-GGTCAGCCGTGAGCAGATGAT-3′) mRNAs. The 5′ end of Dp71 mRNA was PCR amplified using a forward primer in exon G1 (5′-TTGCAGCCATGAGGGAACAG-3′) and a reverse primer in exon 66 (5′-GGACACGGATCCTCCCTGTTCG-3′). Full-length Dp71 mRNA was PCR amplified using the same forward primer in exon G1 and a reverse primer in exon 79 (5′-ATCATCTGCCATGTGGAAAAG-3′). As a loading control, glyceraldehyde 3-phosphate dehydrogenase (GAPDH) mRNA was amplified by PCR^39^. All PCR amplifications were performed in a total volume of 10 µl, containing 1 µl of cDNA, 1 µl of 10 × ExTaq buffer (Takara Bio, Inc., Shiga, Japan), 0.25 U of ExTaq polymerase (Takara Bio, Inc.), 500 nM of each primer, and 250 µM dNTPs (Takara Bio, Inc.), on a Mastercycler Gradient PCR machine (Eppendorf, Hamburg, Germany). The amplification protocol consisted of an initial denaturation at 94 °C for 3 min, followed by 30 cycles (18 for GAPDH) of denaturation at 94 °C for 0.5 min, annealing at 59 °C for 0.5 min, and extension at 72 °C for 1 min. PCR-amplified products were electrophoresed using DNA 1000 or 7500 LabChip kits on an Agilent 2100 Bioanalyzer (Agilent Technologies, Santa Clara, CA, USA). For sequencing, PCR-amplified products were subcloned into the pT7 blue T vector (Novagen, Inc., San Diego, CA, USA). Full length Dp71 was sequenced by two-step sequencing as described^8^. Sequencing by the Sanger method was performed by FASMAC Co., Ltd. (Atsugi, Japan) using an Applied Biosystems Big Dye Terminator V3.1 (Carlsbad, CA, USA).

### Protein analysis

#### Expression of Dp71 and Dp71ab

Plasmids expressing Dp71 and Dp71ab were constructed by inserting the coding sequences of Dp71 and Dp71ab, respectively (Supplementary Table 1), into the mammalian expression vector pcDNA3 with a CMV promoter (Invitrogen, Thermo Fisher Scientific Inc.) by FASMAC Co., Ltd, as described^40^. AGS cells (4 × 10^5^) grown to 80% confluency on six-well culture dishes were transfected with 2 μg of the synthesized plasmids in 3 μL Lipofectamine 3000 (Thermo Fischer Scientific). After incubation for 24 h, the cells were harvested, lysed in RIPA buffer (Cell Signaling Technology Inc., Danvers, MA, USA) containing protease inhibitors and sonicated. After incubation on ice for 20 min, the lysates were centrifuged at 12,000*g* for 20 min to remove insoluble material. The protein concentrations of the cell lysates were determined using QUBIT Protein Assay kits (Thermo Fisher Scientific, Inc.).

#### Western blotting

Lysates of human satellite cells were obtained from ScienCell Research Laboratories (Carlsbad, CA, USA; catalogue no. 3516). These lysates, as well as lysates of cells transfected with plasmids expressing Dp71ab and Dp71 (control), were analyzed by western blotting. Briefly, lysates containing 20 μg protein were mixed 3:1 with Laemmli Sample Buffer (Bio-Rad Laboratories, Inc., Hercules, CA, USA) and boiled for 5 min. These samples and protein size markers (PRECISION PLUS PROTEIN Dual Color Standards; Bio-Rad Laboratories, Inc.) were loaded onto MINI-PROTEAN TGX Precast Gels 4–20% (Bio-Rad Laboratories, Inc.). Following electrophoresis, the proteins were transferred to PVDF Transfer Membranes (GE Healthcare, Little Chalfont, UK). The membranes were blocked with 2% ECL Prime Blocking Reagent (GE Healthcare) and incubated overnight with rabbit antibodies against the C-terminal domain of human dystrophin, either ab15277 (C-terminal amino acids from 3661 to 3777, Abcam, Cambridge, UK) diluted 1:500 or 7A10 (C-terminal amino acids from 3200 to 3684, Santa Cruz Biotechnology, Inc., Santa Cruz, CA, USA) diluted 1:100. As a loading control, the membranes were also incubated with mouse antibody against β-actin (C4, Santa Cruz Biotechnology, Inc.) diluted 1:1000. The membranes were washed and incubated with anti-rabbit IgG (GE Healthcare) or anti-mouse IgG (GE Healthcare) and immunoreactive bands were detected with ECL Select Western Blotting Detection Reagent (GE Healthcare).

### Cell proliferation assay

#### CCK8 assay

Cell proliferation was analyzed by Cell Counting Kit-8 (CCK-8, Dojindo, MD, USA).

Myoblasts (1 × 10^4^/well) were plated in triplicate in 96-well plates. After 24 h, the cells were transfected with 100 ng of pcDNA3 containing the coding sequence for Dp71 or Dp71ab or no insert in 0.3 μL Lipofectamine 3000 (Thermo Fischer Scientific), according to the manufacturer’s protocol. Three hours later, the media were replaced by DMEM. At 24, 48, and 72 h after transfection, 10 μL of CCK-8 solution were added to each well, and the absorbance of each well at 450 nm was determined using a microplate reader (ARVO X3, PerkinElmer). The results shown are representative of three identical experiments.

#### Cell number assay

Cell proliferation was analyzed by counting cells under a microscope. Myoblasts (1 × 10^3^/well) were plated in triplicate in 96 well plates. After 24 h, the cells were transfected with 10 ng of pcDNA3 containing the coding sequence for Dp71, or Dp71ab or no insert in 0.1 μL Lipofectamine 3000 (Thermo Fischer Scientific) according to the manufacturer’s protocol. Cells in a microscopic field of each well captured by the 4 × plan fluor lens of a BZ‐X710 fluorescence microscope (Keyence, Osaka, Japan) were counted 0, 24, 48, and 72 h after transfection and analyzed using BZ-X analytic software. The same area of each well was analyzed at each time point. The numbers of cells were reported as the average of three wells containing the same cell populations. The results shown are representative of three identical experiments.

### Statistical analyses

All assays were repeated 3 times to ensure reproducibility. Results reported as mean ± SE were analyzed by one-way ANOVA and LSD. Results reported as means ± SD were analyzed by ANOVA for comparisons of three or more groups and Student’s t-tests for comparisons between two groups. All statistical analyses were performed using SPSS software (version 17.0; SPSS Inc., Chicago, IL, USA), with P < 0.05 considered statistically significant.

## Supplementary information

Supplementary Figures.

Supplementary Table 1.

## References

[1] Muntoni F, Torelli S, Ferlini A (2003). Dystrophin and mutations: One gene, several proteins, multiple phenotypes. Lancet Neurol..

[2] Ahn AH, Kunkel LM (1993). The structural and functional diversity of dystrophin. Nat. Genet..

[3] Massourides E (2015). Dp412e: A novel human embryonic dystrophin isoform induced by BMP4 in early differentiated cells. Skelet. Muscle.

[4] Bar S (1990). A novel product of the Duchenne muscular dystrophy gene which greatly differs from the known isoforms in its structure and tissue distribution. Biochem. J..

[5] Rapaport D (1992). Characterization and cell type distribution of a novel, major transcript of the Duchenne muscular dystrophy gene. Differentiation.

[6] Tadayoni R, Rendon A, Soria-Jasso LE, Cisneros B (2012). Dystrophin Dp71: The smallest but multifunctional product of the Duchenne muscular dystrophy gene. Mol. Neurobiol..

[7] Aragon J (2018). Dystrophin Dp71 isoforms are differentially expressed in the mouse brain and retina: Report of new alternative splicing and a novel nomenclature for Dp71 isoforms. Mol. Neurobiol..

[8] Rani AQM (2019). Identification of the shortest splice variant of Dp71, together with five known variants, in glioblastoma cells. Biochem. Biophys. Res. Commun..

[9] Marquez FG (2003). Differential expression and subcellular distribution of dystrophin Dp71 isoforms during differentiation process. Neuroscience.

[10] Sarig R (1999). Targeted inactivation of Dp71, the major non-muscle product of the DMD gene: Differential activity of the Dp71 promoter during development. Hum. Mol. Genet..

[11] Villarreal-Silva M, Centeno-Cruz F, Suarez-Sanchez R, Garrido E, Cisneros B (2011). Knockdown of dystrophin Dp71 impairs PC12 cells cycle: Localization in the spindle and cytokinesis structures implies a role for Dp71 in cell division. PLoS ONE.

[12] Garcia-Tovar CG (2002). Dystrophin isoform Dp7l is present in lamellipodia and focal complexes in human astrocytoma cells U-373 MG. Acta Histochem..

[13] Tan S (2016). Knocking down Dp71 expression in A549 cells reduces its malignancy in vivo and in vitro. Cancer Investig..

[14] Tan S (2016). Decreased Dp71 expression is associated with gastric adenocarcinoma prognosis. Oncotarget.

[15] Mauduit O (2019). Recurrent DMD deletions highlight specific role of Dp71 isoform in soft-tissue sarcomas. Cancers.

[16] Ruggieri S (2019). Dp71 expression in human glioblastoma. Int. J. Mol. Sci..

[17] Aragon J (2011). Characterization of Dp71Δ(78–79), a novel dystrophin mutant that stimulates PC12 cell differentiation. J. Neurochem..

[18] Rodriguez-Munoz R (2008). Neuronal differentiation modulates the dystrophin Dp71d binding to the nuclear matrix. Biochem. Biophys. Res. Commun..

[19] Herrera-Salazar A (2016). Overexpression of mutant dystrophin Dp71[INCREMENT]_78–79_ stimulates cell proliferation. NeuroReport.

[20] Aragon J (2016). Identification of Dp71 isoforms expressed in PC12 cells: Subcellular localization and colocalization with β-dystroglycan and α-syntrophin. J. Mol. Neurosci..

[21] Nishida A (2016). HEK293 cells express dystrophin Dp71 with nucleus-specific localization of Dp71ab. Histochem. Cell Biol..

[22] Bushby K (2010). Diagnosis and management of Duchenne muscular dystrophy, part 1: Diagnosis, and pharmacological and psychosocial management. Lancet Neurol..

[23] Sun C, Serra C, Lee G, Wagner KR (2020). Stem cell-based therapies for Duchenne muscular dystrophy. Exp. Neurol..

[24] Chang NC, Chevalier FP, Rudnicki MA (2016). Satellite cells in muscular dystrophy—lost in polarity. Trends Mol. Med..

[25] Webster C, Blau HM (1990). Accelerated age-related decline in replicative life-span of Duchenne muscular dystrophy myoblasts: Implications for cell and gene therapy. Somat. Cell Mol. Genet..

[26] Sacco A (2010). Short telomeres and stem cell exhaustion model Duchenne muscular dystrophy in mdx/mTR mice. Cell.

[27] Dumont NA, Wang YX, Rudnicki MA (2015). Intrinsic and extrinsic mechanisms regulating satellite cell function. Development.

[28] Meregalli M (2013). Perspectives of stem cell therapy in Duchenne muscular dystrophy. FEBS J..

[29] Aziz A, Sebastian S, Dilworth FJ (2012). The origin and fate of muscle satellite cells. Stem Cell Rev. Rep..

[30] Austin RC, Howard PL, D'Souza VN, Klamut HJ, Ray PN (1995). Cloning and characterization of alternatively spliced isoforms of Dp71. Hum. Mol. Genet..

[31] Romo-Yanez J (2007). Dp71ab/DAPs complex composition changes during the differentiation process in PC12 cells. J. Cell. Biochem..

[32] Rau F (2015). Abnormal splicing switch of DMD's penultimate exon compromises muscle fibre maintenance in myotonic dystrophy. Nat. Commun..

[33] Takeshima Y, Nishio H, Sakamoto H, Nakamura H, Matsuo M (1995). Modulation of in vitro splicing of the upstream intron by modifying an intra-exon sequence which is deleted from the dystrophin gene in dystrophin Kobe. J. Clin. Investig..

[34] Matsuo M, Takeshima Y, Nishio H (2016). Contributions of Japanese patients to development of antisense therapy for DMD. Brain Dev..

[35] Miceli MC, Nelson SF (2016). The case for eteplirsen: Paving the way for precision medicine. Mol. Genet. Metab..

[36] Motohashi N, Shimizu-Motohashi Y, Roberts TC, Aoki Y (2019). Potential therapies using myogenic stem cells combined with bio-engineering approaches for treatment of muscular dystrophies. Cells.

[37] Nagata Y (2017). Interleukin-1beta (IL-1β)-induced notch ligand Jagged1 suppresses mitogenic action of IL-1β on human dystrophic myogenic cells. PLoS ONE.

[38] Nishida A (2011). Chemical treatment enhances skipping of a mutated exon in the dystrophin gene. Nat. Commun..

[39] Thi Tran HT (2005). A G-to-A transition at the fifth position of intron 32 of the dystrophin gene inactivates a splice donor site both in vivo and in vitro. Mol. Genet. Metab..

[40] Kawaguchi T (2018). Detection of dystrophin Dp71 in human skeletal muscle using an automated capillary western assay system. Int. J. Mol. Sci..

