# Supplementary material for: Dystrophin Dp71ab is monoclonally expressed in human satellite cells and enhances proliferation of myoblast cells
Source: Sci Rep. 2020 Oct 13;10:17123. doi: 10.1038/s41598-020-74157-y (PMC7553993; doi:10.1038/s41598-020-74157-y)

# Dystrophin Dp71ab is mono-clonally expressed in human satellite cells and enhances proliferation of myoblast cells

Manal Farea, Abdul Qawee Mahyoob Rani, Kazuhiro Maeta, Hisahide Nishio, Masafumi Matsuo

Supplementary Figure 1 Full gel-image of a part of Figure 1

Lanes marked X were excluded because results were not included in this manuscript

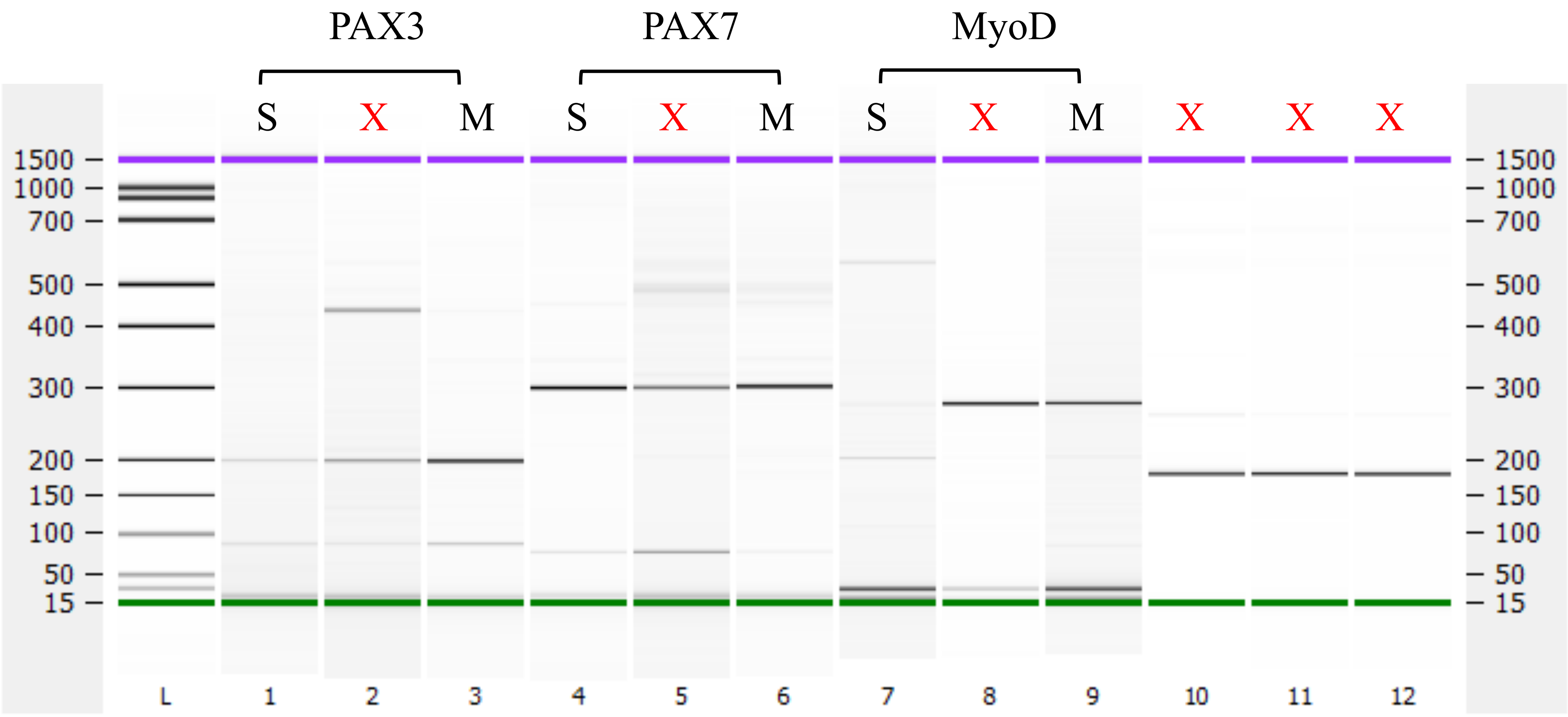

Supplementary Figure 2   Full gel-image of Figure 1

Lanes marked X were excluded because results were not included in this manuscript

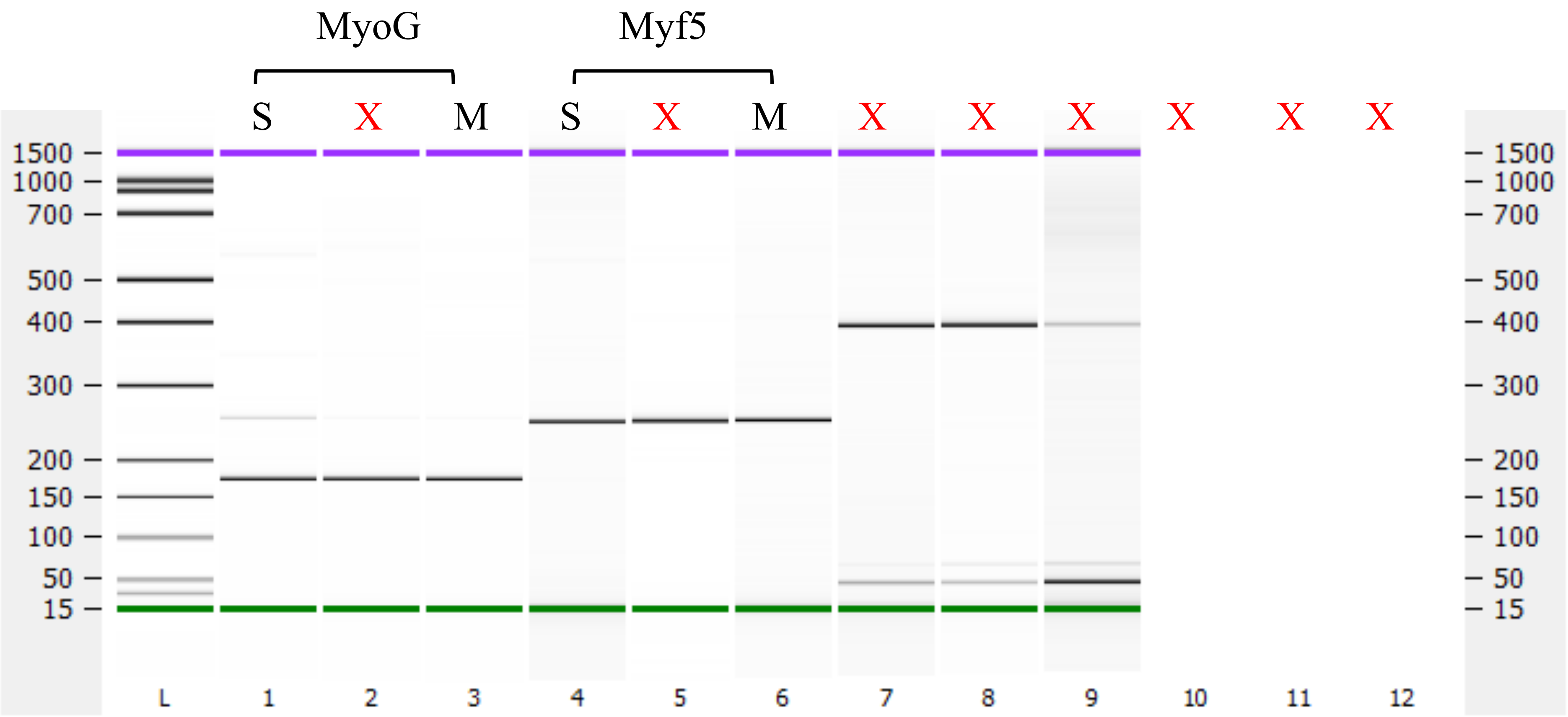

Supplementary Figure 3 Full gel-image of Figure 1

Lanes marked X were excluded because results were not included in this manuscript

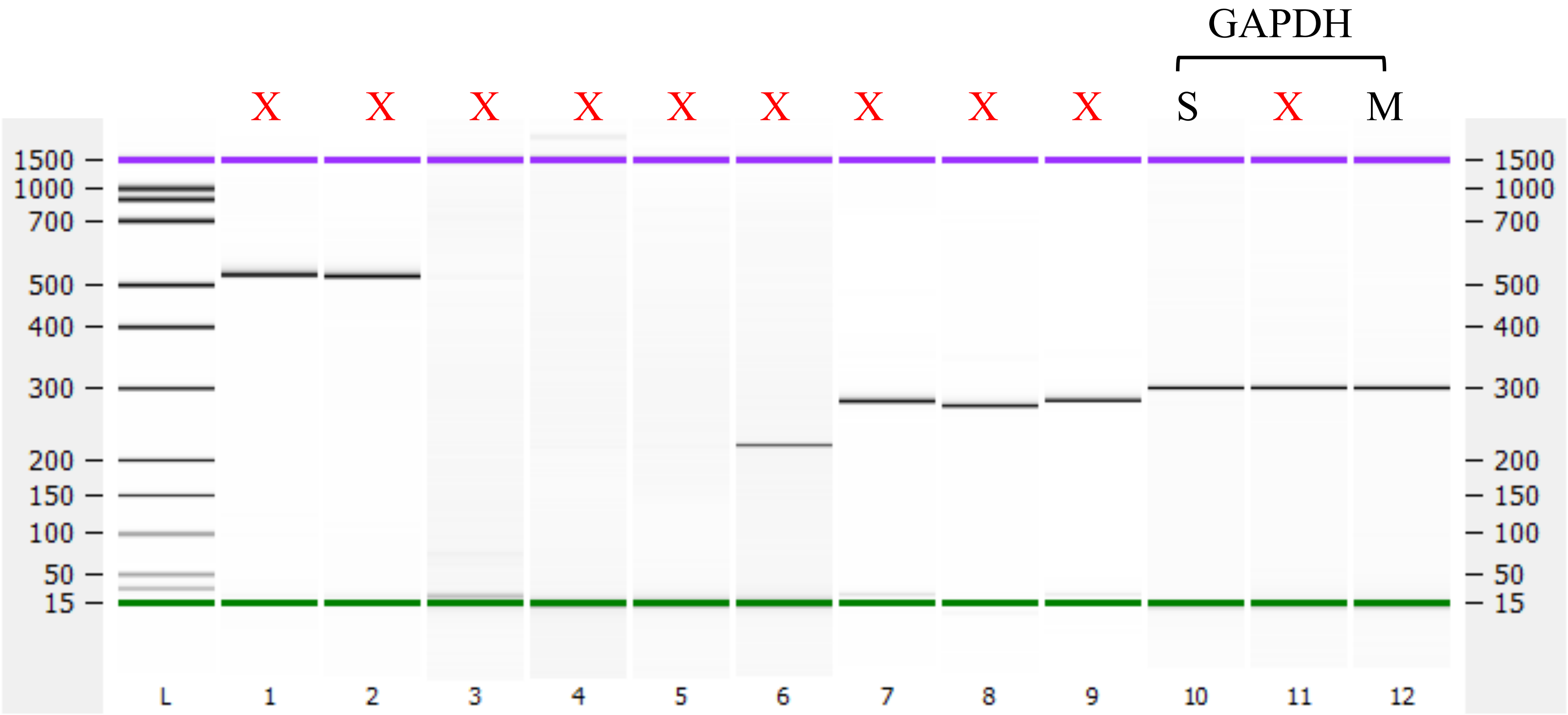

Supplementary Figure 4   Full gel-image of Figure 2B left

Left 3 lanes were cut out.

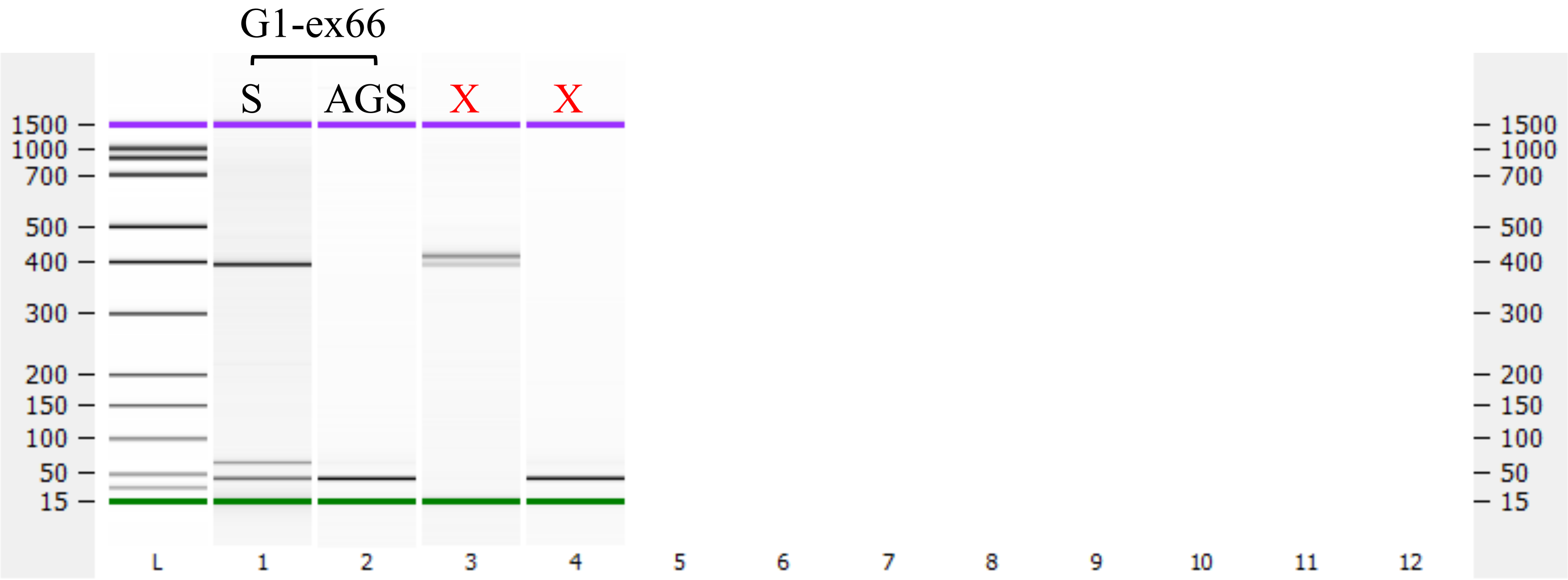

Supplementary Figure 5   Full gel-image of Figure 2B right

Left 3 lanes were cut out.

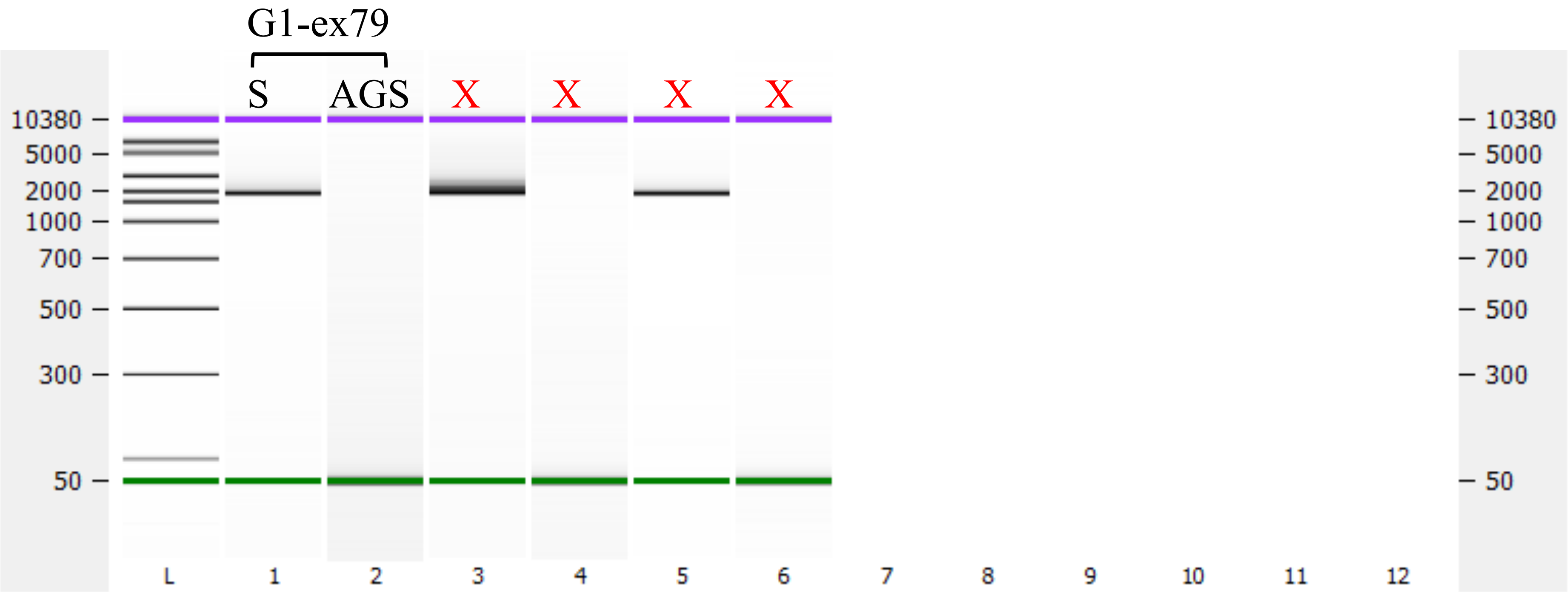

Supplementary Figure 6 Full gel-image of Figure 3A

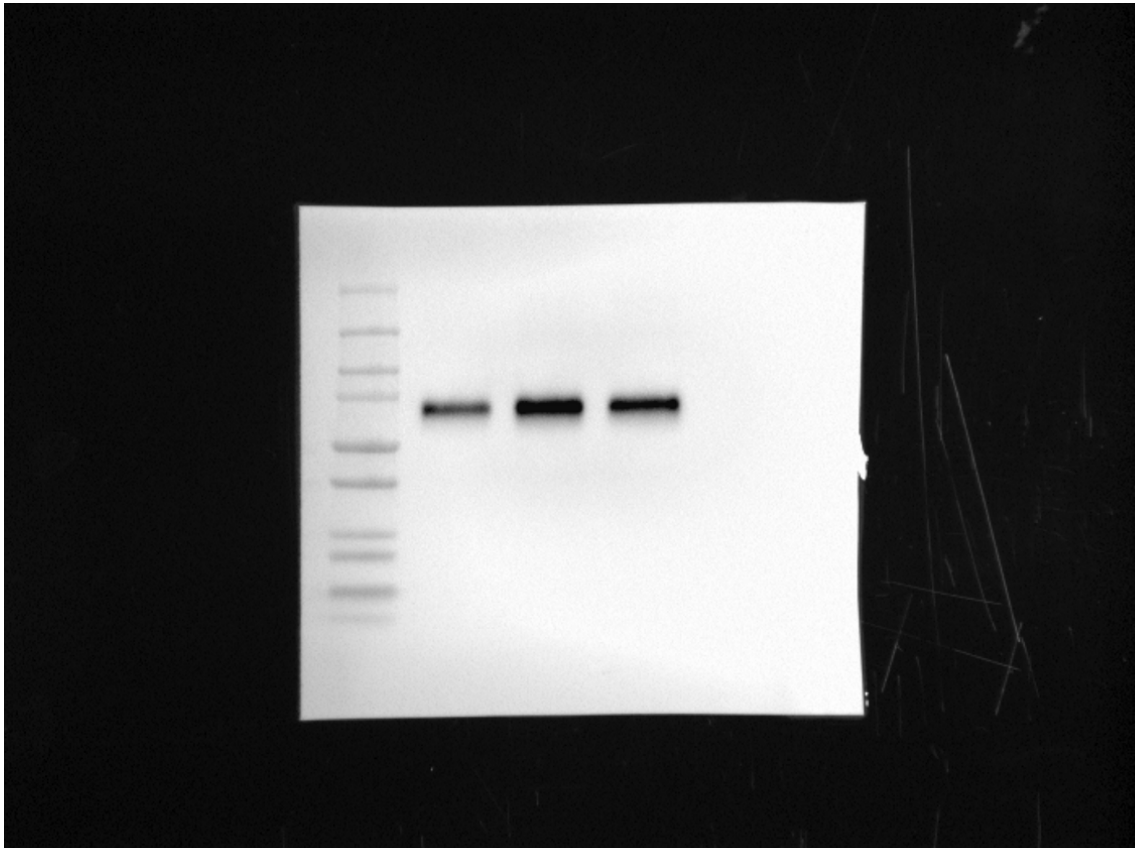

Supplementary Figure 7   Full gel-image of Figure 3B

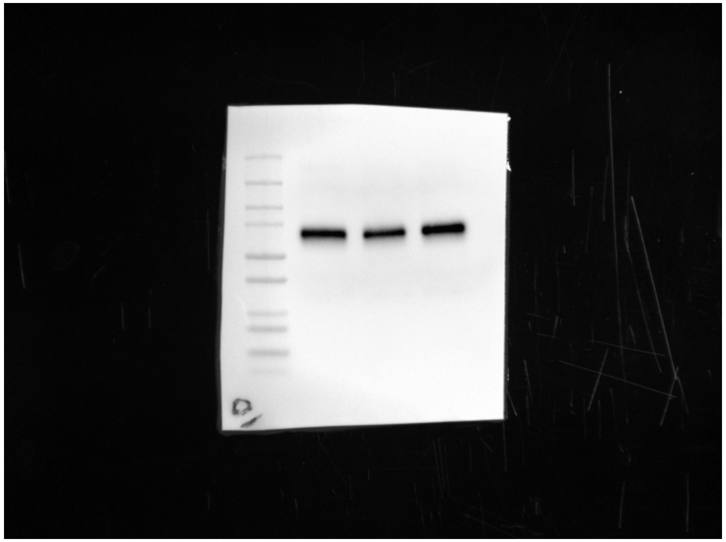

Supplementary Figure 8   Full gel-image of Figure 3A & B

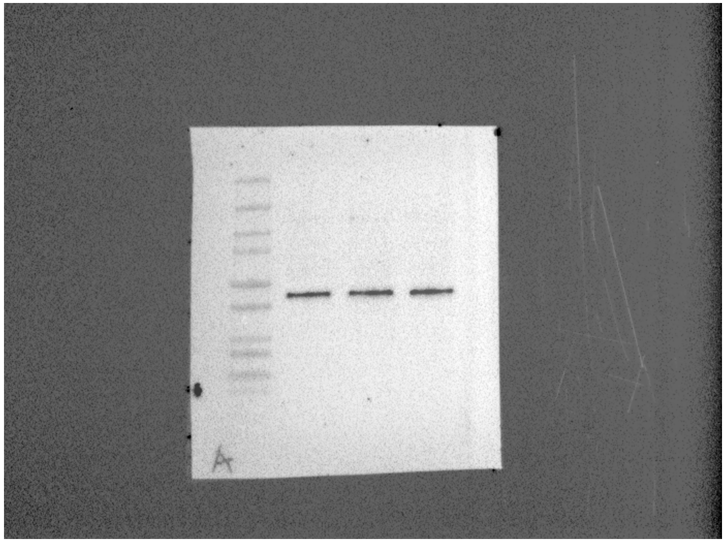

Supplement: Supplementary file 1 — Supplementary Figures. [file 41598_2020_74157_MOESM1_ESM.pdf]
