# Supplementary material for: Dystrophin Dp71ab is monoclonally expressed in human satellite cells and enhances proliferation of myoblast cells
Source: Sci Rep. 2020 Oct 13;10:17123. doi: 10.1038/s41598-020-74157-y (PMC7553993; doi:10.1038/s41598-020-74157-y)
Supplement: Supplementary file 2 — Supplementary Table 1. [file 41598_2020_74157_MOESM2_ESM.docx]

Dystrophin Dp71ab is mono-clonally expressed in human satellite cells and enhances proliferation of myoblast cells

Manal Farea, Abdul Qawee Mahyoob Rani, Kazuhiro Maeta, Hisahide Nishio, Masafumi Matsuo

**Supplementary Table 1 Nucleotide sequences of Dp71 and DP71ab**

Nucleotide sequences of Dp71 and Dp71ab that were inserted into the pcDNA3 are described. Blue and green underlines indicate KpnI and BamHI restriction enzyme recognition sequence, respectively. Translation initiation and stop codons are marked in red.

**A. Dp71**

**GGTACC**ACTTTCGGGGAGCCCGGCGGCTCTGGGAAGCTCACTCCTCCACTCGTACCCACACTCGACCGCGGAGCCCTTGCAGCCATGAGGGAGCAGCTGAAGGGCCATGAGACTCAAACCACTTGTTGGGATCACCCCAAAATGACCGAGTTGTACCAAAGTCTTGCAGACCTGAATAACGTGAGGTTCAGTGCTTATCGGACAGCCATGAAGCTGCGACGACTGCAGAAGGCTTTGTGTCTCGATCTTCTTAGTCTTAGCGCTGCCTGTGACGCACTGGATCAACACAATCTGAAACAAAATGACCAGCCGATGGACATCTTGCAGATCATTAATTGCCTGACTACAATCTACGACCGACTGGAACAGGAACACAATAACCTGGTCAATGTACCCCTTTGCGTCGACATGTGCTTGAACTGGCTTCTGAACGTCTACGACACAGGTCGCACCGGAAGGATTAGGGTTCTCAGCTTCAAGACAGGAATTATTAGCCTTTGCAAGGCCCACCTGGAAGATAAGTACAGATACCTGTTCAAGCAAGTGGCATCAAGTACCGGATTCTGCGATCAGAGAAGGCTCGGGCTCCTTCTGCATGATAGTATCCAGATCCCCAGACAACTGGGCGAGGTAGCATCCTTCGGTGGGTCTAATATCGAGCCATCCGTGCGGTCTTGTTTTCAGTTCGCCAATAACAAACCCGAAATTGAGGCCGCCCTCTTCCTTGACTGGATGCGCCTGGAACCCCAGAGCATGGTGTGGCTGCCCGTGTTGCATCGCGTGGCGGCAGCAGAGACGGCCAAACATCAGGCCAAATGCAATATCTGCAAGGAATGTCCGATCATCGGCTTTAGATATAGATCACTGAAGCACTTTAACTATGATATCTGCCAATCTTGCTTCTTCTCTGGAAGGGTCGCTAAGGGACATAAGATGCATTATCCTATGGTGGAATATTGTACACCGACGACGTCAGGTGAAGATGTGAGAGATTTCGCGAAGGTTCTCAAGAACAAATTCCGCACTAAGCGCTATTTCGCCAAACATCCTCGGATGGGGTATCTGCCTGTACAGACGGTGCTTGAGGGCGATAACATGGAGACTCCAGTCACACTCATTAATTTCTGGCCCGTAGATAGCGCGCCTGCATCATCACCACAATTGTCTCATGACGACACTCATTCCAGAATTGAACATTACGCGTCCCGCCTGGCTGAGATGGAGAACTCTAATGGCTCCTATCTGAATGATTCCATAAGCCCCAACGAATCTATCGATGACGAACATCTTCTGATCCAACACTATTGTCAAAGTCTGAACCAAGATTCCCCTCTGAGTCAACCAAGGAGCCCCGCGCAAATCCTTATTAGCCTGGAGTCCGAAGAGCGCGGGGAGCTCGAGCGCATATTGGCCGACTTGGAAGAAGAGAACAGAAATCTGCAGGCTGAATACGATCGGTTGAAACAACAACACGAACATAAAGGACTGTCTCCATTGCCATCACCGCCTGAAATGATGCCCACCAGCCCACAGTCCCCAAGGGATGCCGAACTGATTGCCGAGGCCAAGCTCCTGCGGCAACATAAAGGACGGCTTGAGGCGCGAATGCAGATATTGGAGGACCATAACAAACAGCTCGAAAGTCAACTGCACCGACTGAGGCAACTTCTTGAACAGCCTCAAGCCGAGGCGAAGGTTAATGGTACAACTGTCTCCAGTCCCTCTACTTCTTTGCAACGGTCTGACAGTTCCCAGCCAATGCTCCTGCGCGTGGTCGGCTCACAGACCTCCGACAGCATGGGAGAGGAGGACCTTCTTAGCCCGCCCCAGGATACCTCTACTGGCCTCGAGGAGGTGATGGAGCAACTGAACAACAGTTTTCCTAGCTCTCGCGGACGCAACACACCCGGCAAACCTATGCGCGAAGACACCATGTGAGAAGTCTTTTCCACATGGCAGATGATTTGGGCAGAGCGATGGAGTCCTTAGTATCAGTCATGACAGATGAAGAAGGAGCAGAATAAATGTTTTACAACTCCTGATTCCCGCATGGTTTTTATAATATTCATACAACAAAGAGGATTAGACAGTAAGAGTTTACAAGAAATAAATCTATATTTTTGTGAAGGGTAGTGGTATTATACTGTAGATTTCAGTAGTTTCTAAGTCTGTTATTGTTTTGTTAACAATGGCAGGTTTTACACGTCTATGCAATTGTACAAAAAAGTTATAAGAAAACTACATGTAAAATCTTGATAGCTAAATAACTTGCCATTTCTTTATATGGAACGCATTTTGGGTTGTTTAAAAATTTATAACAGTTATAAAGAAAGATTGTAAACTAAAGTGTGCTTTATAAAAAAAAGTTGTTTATAAAAACCCCTAAAAACAAAACAAACACACACACACACACATACACACACACACACAAAACTTTGAGGCAGCGCATTGTTTTGCATCCTTTTGGCGTGATATCCATATGAAATTCATGGCTTTTTCTTTTTTTGCATATTAAAGATAAGACTTCCTCTACCACCACACCAAATGACTACTACACACTGCTCATTTGAGAACTGTCAGCTGAGTGGGGCAGGCTTGAGTTTTCATTTCATATATCTATATGTCTATAAGTATATAAATACTATAGTTATATAGATAAAGAGATACGAATTTCTATAGACTGACTTTTTCCATTTTTTAAATGTTCATGTCACATCCTAATAGAAAGAAATTACTTCTAGTCAGTCATCCAGGCTTACCTGCTTGGTCTAGAATGGATTTTTCCCGGAGCCGGAAGCCAGGAGGAAACTACACCACACTAAAACATTGTCTACAGCTCCAGATGTTTCTCATTTTAAACAACTTTCCACTGACAACGAAAGTAAAGTAAAGTATTGGATTTTTTTAAAGGGAACATGTGAATGAATACACAGGACTTATTATATCAGAGTGAGTAATCGGTTGGTTGGTTGATTGATTGATTGATTGATACATTCAGCTTCCTGCTGCTAGCAATGCCACGATTTAGATTTAATGATGCTTCAGTGGAAATCAATCAGAAGGTATTCTGACCTTGTGAACATCAGAAGGTATTTTTTAACTCCCAAGCAGTAGCAGGACGATGATAGGGCTGGAGGGCTATGGATTCCCAGCCCATCCCTGTGAAGGAGTAGGCCACTCTTTAAGTGAAGGATTGGATGATTGTTCATAATACATAAAGTTCTCTGTAATTACAACTAAATTATTATGCCCTCTTCTCACAGTCAAAAGGAACTGGGTGGTTTGGTTTTTGTTGCTTTTTTAGATTTATTGTCCCATGTGGGATGAGTTTTTAAATGCCACAAGACATAATTTAAAATAAATAAACTTTGGGAAAAGGTGTAAGACAGTAGCCCCATCACATTTGTGATACTGACAGGTATCAACCCAGAAGCCCATGAACTGTGTTTCCATCCTTTGCATTTCTCTGCGAGTAGTTCCACACAGGTTTGTAAGTAAGTAAGAAAGAAGGCAAATTGATTCAAATGTTACAAAAAAACCCTTCTTGGTGGATTAGACAGGTTAAATATATAAACAAACAAACAAAAATTGCTCAAAAAAGAGGAGAAAAGCTCAAGAGGAAAAGCTAAGGACTGGTAGGAAAAAGCTTTACTCTTTCATGCCATTTTATTTCTTTTTGATTTTTAAATCATTCATTCAATAGATACCACCGTGTGACCTATAATTTTGCAAATCTGTTACCTCTGACATCAAGTGTAATTAGCTTTTGGAGAGTGGGCTGACATCAAGTGTAATTAGCTTTTGGAGAGTGGGTTTTGTCCATTATTAATAATTAATTAATTAACATCAAACACGGCTTCTCATGCTATTTCTACCTCACTTTGGTTTTGGGGTGTTCCTGATAATTGTGCACACCTGAGTTCACAGCTTCACCACTTGTCCATTGCGTTATTTTCTTTTTCCTTTATAATTCTTTCTTTTTCCTTCATAATTTTCAAAAGAAAACCCAAAGCTCTAAGGTAACAAATTACCAAATTACATGAAGATTTGGTTTTTGTCTTGCATTTTTTTCCTTTATGTGACGCTGGACCTTTTCTTTACCCAAGGATTTTTAAAACTCAGATTTAAAACAAGGGGTTACTTTACATCCTACTAAGAAGTTTAAGTAAGTAAGTTTCATTCTAAAATCAGAGGTAAATAGAGTGCATAAATAATTTTGTTTTAATCTTTTTGTTTTTCTTTTAGACACATTAGCTCTGGAGTGAGTCTGTCATAATATTTGAACAAAAATTGAGAGCTTTATTGCTGCATTTTAAGCATAATTAATTTGGACATTATTTCGTGTTGTGTTCTTTATAACCACCGAGTATTAAACTGTAAATCATAATGTAACTGAAGCATAAACATCACATGGCATGTTTTGTCATTGTTTTCAGGTACTGAGTTCTTACTTGAGTATCATAATATATTGTGTTTTAACACCAACACTGTAACATTTACGAATTATTTTTTTAAACTTCAGTTTTACTGCATTTTCACAACATATCAGACTTCACCAAATATATGCCTTACTATTGTATTATAGTACTGCTTTACTGTGTATCTCAATAAAGCACGCAGTTATGTTACGGATCC

**B. Dp71ab**

**GGTACC**ACTTTCGGGGAGCCCGGCGGCTCTGGGAAGCTCACTCCTCCACTCGTACCCACACTCGACCGCGGAGCCCTTGCAGCCATGAGGGAGCAGCTGAAGGGCCATGAGACTCAAACCACTTGTTGGGATCACCCCAAAATGACCGAGTTGTACCAAAGTCTTGCAGACCTGAATAACGTGAGGTTCAGTGCTTATCGGACAGCCATGAAGCTGCGACGACTGCAGAAGGCTTTGTGTCTCGATCTTCTTAGTCTTAGCGCTGCCTGTGACGCACTGGATCAACACAATCTGAAACAAAATGACCAGCCGATGGACATCTTGCAGATCATTAATTGCCTGACTACAATCTACGACCGACTGGAACAGGAACACAATAACCTGGTCAATGTACCCCTTTGCGTCGACATGTGCTTGAACTGGCTTCTGAACGTCTACGACACAGGTCGCACCGGAAGGATTAGGGTTCTCAGCTTCAAGACAGGAATTATTAGCCTTTGCAAGGCCCACCTGGAAGATAAGTACAGATACCTGTTCAAGCAAGTGGCATCAAGTACCGGATTCTGCGATCAGAGAAGGCTCGGGCTCCTTCTGCATGATAGTATCCAGATCCCCAGACAACTGGGCGAGGTAGCATCCTTCGGTGGGTCTAATATCGAGCCATCCGTGCGGTCTTGTTTTCAGTTCGCCAATAACAAACCCGAAATTGAGGCCGCCCTCTTCCTTGACTGGATGCGCCTGGAACCCCAGAGCATGGTGTGGCTGCCCGTGTTGCATCGCGTGGCGGCAGCAGAGACGGCCAAACATCAGGCCAAATGCAATATCTGCAAGGAATGTCCGATCATCGGCTTTAGATATAGATCACTGAAGCACTTTAACTATGATATCTGCCAATCTTGCTTCTTCTCTGGAAGGGTCGCTAAGGGACATAAGATGCATTATCCTATGGTGGAATATTGTACACCGACGACGTCAGGTGAAGATGTGAGAGATTTCGCGAAGGTTCTCAAGAACAAATTCCGCACTAAGCGCTATTTCGCCAAACATCCTCGGATGGGGTATCTGCCTGTACAGACGGTGCTTGAGGGCGATAACATGGAGACTCCTGCATCATCACCACAATTGTCTCATGACGACACTCATTCCAGAATTGAACATTACGCGTCCCGCCTGGCTGAGATGGAGAACTCTAATGGCTCCTATCTGAATGATTCCATAAGCCCCAACGAATCTATCGATGACGAACATCTTCTGATCCAACACTATTGTCAAAGTCTGAACCAAGATTCCCCTCTGAGTCAACCAAGGAGCCCCGCGCAAATCCTTATTAGCCTGGAGTCCGAAGAGCGCGGGGAGCTCGAGCGCATATTGGCCGACTTGGAAGAAGAGAACAGAAATCTGCAGGCTGAATACGATCGGTTGAAACAACAACACGAACATAAAGGACTGTCTCCATTGCCATCACCGCCTGAAATGATGCCCACCAGCCCACAGTCCCCAAGGGATGCCGAACTGATTGCCGAGGCCAAGCTCCTGCGGCAACATAAAGGACGGCTTGAGGCGCGAATGCAGATATTGGAGGACCATAACAAACAGCTCGAAAGTCAACTGCACCGACTGAGGCAACTTCTTGAACAGCCTCAAGCCGAGGCGAAGGTTAATGGTACAACTGTCTCCAGTCCCTCTACTTCTTTGCAACGGTCTGACAGTTCCCAGCCAATGCTCCTGCGCGTGGTCGGCTCACAGACCTCCGACAGCATGGGAGAGGAGGACCTTCTTAGCCCGCCCCAGGATACCTCTACTGGCCTCGAGGAGGTGATGGAGCAACTGAACAACAGTTTTCCTAGCTCTCGCGGACATAATGTGGGATCTCTGTTTCACATGGCCGATGACCTGGGCAGGGCCATGGAATCTCTGGTGTCAGTGATGACTGATGAGGAAGGTGCTGAGTGAATGTTTTACAACTCCTGATTCCCGCATGGTTTTTATAATATTCATACAACAAAGAGGATTAGACAGTAAGAGTTTACAAGAAATAAATCTATATTTTTGTGAAGGGTAGTGGTATTATACTGTAGATTTCAGTAGTTTCTAAGTCTGTTATTGTTTTGTTAACAATGGCAGGTTTTACACGTCTATGCAATTGTACAAAAAAGTTATAAGAAAACTACATGTAAAATCTTGATAGCTAAATAACTTGCCATTTCTTTATATGGAACGCATTTTGGGTTGTTTAAAAATTTATAACAGTTATAAAGAAAGATTGTAAACTAAAGTGTGCTTTATAAAAAAAAGTTGTTTATAAAAACCCCTAAAAACAAAACAAACACACACACACACACATACACACACACACACAAAACTTTGAGGCAGCGCATTGTTTTGCATCCTTTTGGCGTGATATCCATATGAAATTCATGGCTTTTTCTTTTTTTGCATATTAAAGATAAGACTTCCTCTACCACCACACCAAATGACTACTACACACTGCTCATTTGAGAACTGTCAGCTGAGTGGGGCAGGCTTGAGTTTTCATTTCATATATCTATATGTCTATAAGTATATAAATACTATAGTTATATAGATAAAGAGATACGAATTTCTATAGACTGACTTTTTCCATTTTTTAAATGTTCATGTCACATCCTAATAGAAAGAAATTACTTCTAGTCAGTCATCCAGGCTTACCTGCTTGGTCTAGAATGGATTTTTCCCGGAGCCGGAAGCCAGGAGGAAACTACACCACACTAAAACATTGTCTACAGCTCCAGATGTTTCTCATTTTAAACAACTTTCCACTGACAACGAAAGTAAAGTAAAGTATTGGATTTTTTTAAAGGGAACATGTGAATGAATACACAGGACTTATTATATCAGAGTGAGTAATCGGTTGGTTGGTTGATTGATTGATTGATTGATACATTCAGCTTCCTGCTGCTAGCAATGCCACGATTTAGATTTAATGATGCTTCAGTGGAAATCAATCAGAAGGTATTCTGACCTTGTGAACATCAGAAGGTATTTTTTAACTCCCAAGCAGTAGCAGGACGATGATAGGGCTGGAGGGCTATGGATTCCCAGCCCATCCCTGTGAAGGAGTAGGCCACTCTTTAAGTGAAGGATTGGATGATTGTTCATAATACATAAAGTTCTCTGTAATTACAACTAAATTATTATGCCCTCTTCTCACAGTCAAAAGGAACTGGGTGGTTTGGTTTTTGTTGCTTTTTTAGATTTATTGTCCCATGTGGGATGAGTTTTTAAATGCCACAAGACATAATTTAAAATAAATAAACTTTGGGAAAAGGTGTAAGACAGTAGCCCCATCACATTTGTGATACTGACAGGTATCAACCCAGAAGCCCATGAACTGTGTTTCCATCCTTTGCATTTCTCTGCGAGTAGTTCCACACAGGTTTGTAAGTAAGTAAGAAAGAAGGCAAATTGATTCAAATGTTACAAAAAAACCCTTCTTGGTGGATTAGACAGGTTAAATATATAAACAAACAAACAAAAATTGCTCAAAAAAGAGGAGAAAAGCTCAAGAGGAAAAGCTAAGGACTGGTAGGAAAAAGCTTTACTCTTTCATGCCATTTTATTTCTTTTTGATTTTTAAATCATTCATTCAATAGATACCACCGTGTGACCTATAATTTTGCAAATCTGTTACCTCTGACATCAAGTGTAATTAGCTTTTGGAGAGTGGGCTGACATCAAGTGTAATTAGCTTTTGGAGAGTGGGTTTTGTCCATTATTAATAATTAATTAATTAACATCAAACACGGCTTCTCATGCTATTTCTACCTCACTTTGGTTTTGGGGTGTTCCTGATAATTGTGCACACCTGAGTTCACAGCTTCACCACTTGTCCATTGCGTTATTTTCTTTTTCCTTTATAATTCTTTCTTTTTCCTTCATAATTTTCAAAAGAAAACCCAAAGCTCTAAGGTAACAAATTACCAAATTACATGAAGATTTGGTTTTTGTCTTGCATTTTTTTCCTTTATGTGACGCTGGACCTTTTCTTTACCCAAGGATTTTTAAAACTCAGATTTAAAACAAGGGGTTACTTTACATCCTACTAAGAAGTTTAAGTAAGTAAGTTTCATTCTAAAATCAGAGGTAAATAGAGTGCATAAATAATTTTGTTTTAATCTTTTTGTTTTTCTTTTAGACACATTAGCTCTGGAGTGAGTCTGTCATAATATTTGAACAAAAATTGAGAGCTTTATTGCTGCATTTTAAGCATAATTAATTTGGACATTATTTCGTGTTGTGTTCTTTATAACCACCGAGTATTAAACTGTAAATCATAATGTAACTGAAGCATAAACATCACATGGCATGTTTTGTCATTGTTTTCAGGTACTGAGTTCTTACTTGAGTATCATAATATATTGTGTTTTAACACCAACACTGTAACATTTACGAATTATTTTTTTAAACTTCAGTTTTACTGCATTTTCACAACATATCAGACTTCACCAAATATATGCCTTACTATTGTATTATAGTACTGCTTTACTGTGTATCTCAATAAAGCACGCAGTTATGTTAC**GGATCC**
